# A mosaic karyotype of 45,X/46,X,psu idic(Y)(q12) in a ten-year-old boy: integrating high-throughput sequencing with cytogenetic technique for precise diagnosis and genetic counselling

**DOI:** 10.1186/s12887-023-03872-y

**Published:** 2023-03-04

**Authors:** Hui Yin, Hua Xie, Jizhen Zou, Xue Ye, Ying Liu, Cai He, Shaofang Shangguan, Haoran Liu, Xiaoli Chen, Xiaobo Chen

**Affiliations:** 1 Department of Endocrinology, Affiliated Children’s Hospital of Capital Institute of Pediatrics, No.2, Yabao Road, Chaoyang District, Beijing, 100020 China; 2 Department of Medical Genetics, Capital Institute of Pediatrics, Room 616, No.2, Yabao Road, Chaoyang District, Beijing, 100020 China; 3 Department of Pathology, Affiliated Children’s Hospital of Capital Institute of Pediatrics, Beijing, China

**Keywords:** Isodicentric Y chromosome, Mosaicism, High-throughput sequencing, Precise diagnosis, Genetic counselling

## Abstract

**Background:**

Isodicentric Y chromosome (idic(Y)) is the most commonly reported aberration of the human Y chromosome, which is an important cause of abnormal sexual development. The breakpoints of isodicentric Y chromosome mostly occurred in Yq11.2 and Yp11.3, however, the breakpoints in Yq12 are relatively rare.

**Case presentation:**

We described a 10-year-old boy presenting hypospadias, micropenis and short stature, as well as unilateral cryptorchidism without normal testicular seminiferous tubules structure by biopsy. Whole exome sequencing didn’t find any pathogenic/likely pathogenic variants related to phenotypes of this patient. Copy number variation sequencing showed the duplication of whole Y chromosome. Subsequently, karyotyping and FISH analyses demonstrated his genetic diagnosis was mosaic 45,X[8]/46,X,psu idic(Y)(q12)[32], with the breakpoint in Yq12.

**Conclusions:**

Our case proved that it would be beneficial to integrate high-throughput sequencing with cytogenetic technique for precise diagnosis, treatment and genetic counselling.

## Background

The Y chromosome is one of the smallest chromosomes in human male, consisting of a short (Yp) arm and a long (Yq) one. The genes on the Y chromosome such as sex-determining gene *SRY* and spermatogenesis genes (azoospermia factor, *AZF*) are important for male sex determination and spermatogenesis [1]. Isodicentric Y chromosome (idic(Y)) is the most common Y chromosome aberration, and it consists of two identical arms with an axis of symmetry lying between two centromeres [2]. The unstable genomic structures of Y chromosome also occur in different cell lines, and consequently, most reported patients have alternate cell lines, including 45,X cell line [3]. The resulting phenotypes of the affected cases vary from healthy infertile males, females with or without Turner syndrome to individuals with ambiguous genitalia or with mixed gonadal dysgenesis, depending on the proportion of mosaics as well as the location of the breakpoints [4].

Here, we detailedly described a patient with short stature, hypospadias, micropenis and unilateral cryptorchidism without normal structure of testicular seminiferous tubules. We finished the accurate genetic diagnosis of the mosaic karyotype 45,X[8]/46,X,psu idic(Y)(q12)[32], combining high-throughput sequencing and cytogenetic tests.

## Case presentation

A 10-year-old boy was referred to Affiliated Children’s Hospital of Capital Institute of Pediatrics due to complaints of short stature and micropenis. His mother and father had normal karyotypes. No family history of sterility or abortion was reported. The mother's and the father's height were 171 cm and 175 cm, respectively.

**Fig. 1:**
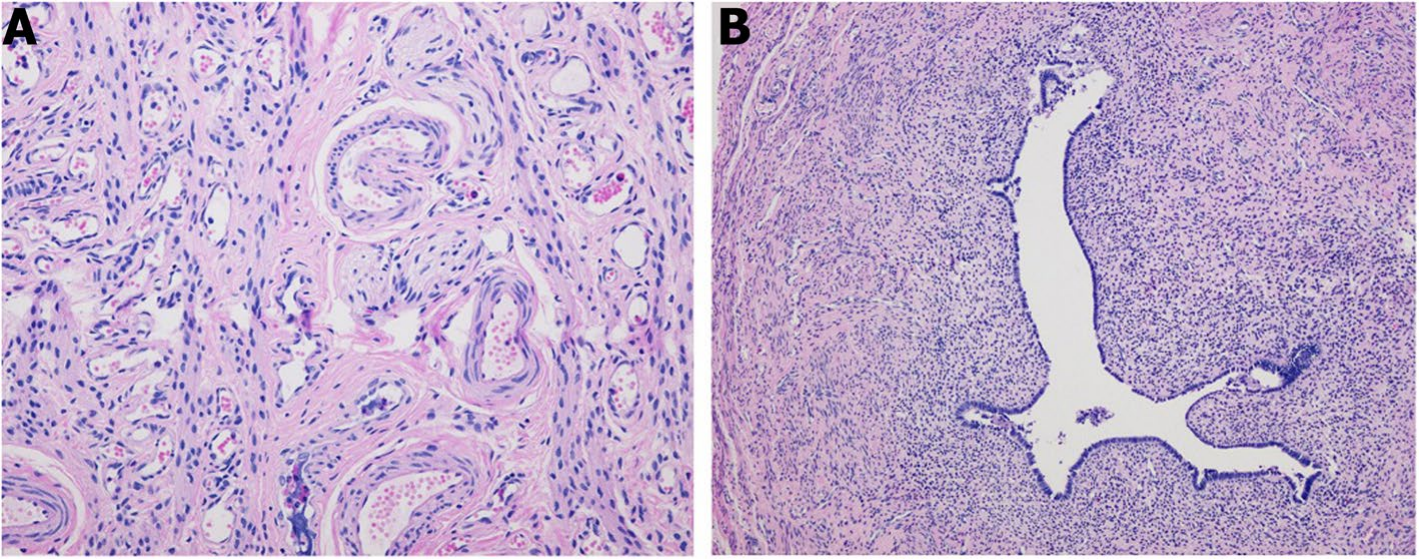
Testicular biopsy with immature testicular (**A**) and epididymal (**B**)

The patient underwent a C-section at 37 weeks because of fetal distress. His birth weight and height were 2960 g and 49 cm respectively. He was diagnosed as hypoxic ischemic encephalopathy (HIE) at birth, and showed borderline developmental delay since he was six months old. He suffered from epilepsy at age of 11 months. He was diagnosed as hypospadias, unilateral cryptorchidism and underwent left radical orchiectomy and urethroplasty when he was 13 months old. The biopsy of the left testicular didn’t show normal structure of seminiferous tubules. Instead, a number of fibrous connective tissue and new blood vessel presented (Fig. 1A). In addition, no mature structure of epididymal duct was seen in the left epididymidis with the full of immature mesenchymal cells (Fig. 1B).

The growth velocity was 3-4 cm per year since he was 5 years old. Physical examination at age of 10 showed a height of 129 cm (-2 SDS) and weight of 12 kg (-1.5 SDS) with proportional short stature. Genitourinary checkup revealed Prader class 5 genitalia, with approximately 4 cm-size penis. No palpable gonad was detected bilaterally in the scrotum. Ultrasound of the gonads showed right testis in the groin area, with a size of 1.39 × 0.63 cm. The baseline level of anti-mullerian hormone and inhibin B indicated dysfunction of sertoli cells (Table 1). Also, the level of testosterone concentration was relatively low. Further examination of these hormones after HCG and GnRHa stimulation demonstrated normal pituitary reaction and poor function of leydig cell, respectively (Table 1).

**Table 1:** The results of hormone test

	––	**LH (IU/L)**	**FSH (IU/L)**	**Testosterone (nmol/l)**	**Anti-Mullerian hormone (ng/ml)**	**Inhibin B (pg/ml)**
**Baseline**		0.94 (0–7.8)	2.18 (0–4.6)	0.087 (0–2.37)	2.3 (0.77–14.5)	2.1 (41–328)
**HCG stimulating**	-	-	-	0.813 (> 3.5)		
**GnRHa stimulating**	30 min	2.57	3.75	-		
	60 min	3.63	5.35	-		
	90 min	4.09	6.45	-		

### Whole exome sequencing (WES) and copy number variation sequencing (CNV-Seq)

**Fig. 2:**
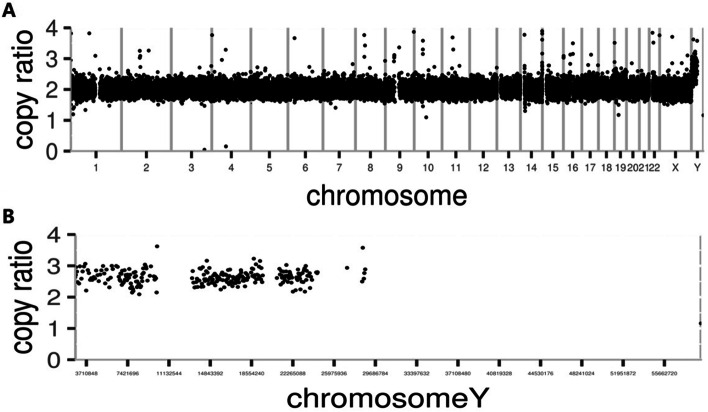
The results of CNV-Seq. **A** CNV-Seq analyses for all the chromosomes. **B** CNV-Seq analyses for Y chromosome

The phenotypes including “short stature”, “micropenis”, “hypospadias” and “epilepsy” were used to filter the related genetic variant, and no pathogenic/likely pathogenic variants was detected from WES according to American College of Medical Genetics and Genomics (ACMG) guidelines. To identify copy number variations (CNVs), CNV-Seq library was constructed using KAPA Library Preparation Kit. An in-house pipeline was applied to map and call CNVs using CNV-kit [5], a health male with normal karyotype was used as control sample. The CNV-Seq showed duplication of whole Y chromosome with the ratio between 2 to 4, and no other pathogenic/likely pathogenic CNVs in autosomes or X chromosome identified (Fig. 2A, B).

### Cytogenetic and FISH analysis

**Fig. 3:**
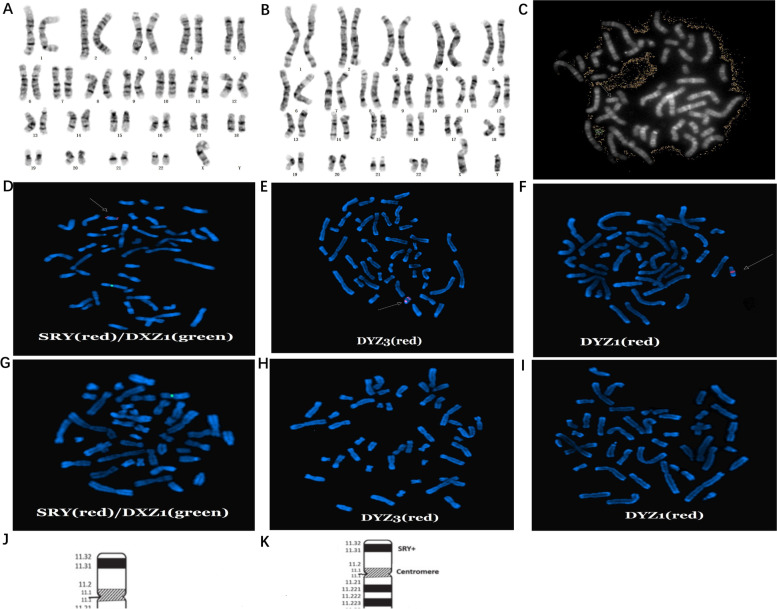
The karyotype (**A–C**), metaphase FISH analyses (**D–I**) for the patient, and diagram of Y chromosome (**J**, **K**). **D-F** FISH signals on cell line of 46,X,idic(Y) karyotype. **D** SRY shown two red signals and DXZ1 shown only one green signal, respectively. **E** Two red DYZ3 signals revealed the inactive Y centromere and one red signal revealed the active Y centromere. **F** DYZ1 signal (red) presenting Yq12 heterochromatin shown minor and near the region of the Yq11.23 **G-I** FISH signals on cell line of 45,X karyotype. **G** Only one DXZ1(green) signal. **H**, **I** No DYZ3 or DYZ1 signal was observed, respectively.** J** Diagram of a normal Y chromosome. **K** Diagram of a isodicentric Y chromosome idic(Y)(q12). The breakpoint (arrow) is located in Yq12 (long arm), with a duplication of short arm, centromere, and proximal long arm and deletion of most part of Yq12 material

High-resolution G-banding (550–650 bands) and FISH using standard techniques were performed to reveal the precise structural arrangement of Y chromosome and mosaics proportion. Chromosomal analysis of peripheral lymphocytes revealed the presence of two cell lines. In 7 of 50 (14%) cells analyzed metaphases, a numerically abnormal karyotype was observed: 45,X (Fig. 3A). In 43 of 50 (86%) metaphases cells, a suspected dicentric Y chromosome was seen: 46,X,psu dic(Y)(q12) (Fig. 3B). In addition, absence of Yq12 heterochromatin was shown in Q-banding (Fig. 3C), with suspected breakpoint in Yq12. Therefore, the karyotype was preliminarily determined as mos 45,X[7]/46,X,psu dic(Y)(q12)[43]. FISH was performed with probes specific for the following regions: Yq12 heterochromatin [DYZ1 (Vysis; Abbott Molecular, Inc., Des Plaines, IL, USA)], Y centromere [DYZ3 (Vysis)], sex region of the Y (Yp11.31) and X centromere [SRY/DXZ1 (Vysis)]. In the karyotype of 46,X,psu idic(Y), FISH analyses revealed two signals of SRY and DYZ3, but a small signal of DYZ1 which confirmed the breakpoint in Yq12 and deletion of most part of Yq12. Combining with the cytogenetic and FISH analysis, we demonstrating final karyotype with 45,X[8]/46,X,psu idic(Y).ish psu idic(Y)(q12)(SRY +  + ,DYZ3 +  + ,DYZ1 +)[32]. The schematic diagram of isodicentric Y chromosome idic(Y)(q12) was illuminated in Fig. 3J, K.

## Discussion and conclusions

Due to the instability of isodicentric Y chromosome during cell division, it is likely to generate different cell lines. Several types of cell lines are found in reported patients with idic(Y), most commonly including 45,X cell line (95% of cases) [3]. Cases with mosaic 45,X/46,X,idic(Y) usually have very wide and variable phenotypes [6, 7]. Among these symptoms, ambiguous genitalia and short stature were most common.

We reported a new case presenting multiple phenotypes including short stature, hypospadias, micropenis and unilateral cryptorchidism. The karyotype of this patient showed two different cell lines: 45,X and 46,X,idic(Y), with the breakpoint was determined in Yq12. As we known, the phenotypes resulting from isodicentric Y chromosomes depends on the proportion of mosaics as well as the location of the breakpoints [4]. In order to explore the influence of mosaics with 45,X/46,X,dic(Y)(q12), we reviewed their phenotypes previously published and found that were variable (Table 2). In our case, in addition to the common phenotypes (ambiguous sexual development and short stature), testicular biopsy of the boy was proven to be immature tissues without normal seminiferous tubules structure. For his testicular function, the results of laboratory examination such as anti-mullerian hormone and inhibin B indicated poor function of his sertoli cells. This means that he is likely to be sterile in adulthood, as reported in previous literatures [8–10]. Moreover, compared with the low level of testosterone, the levels of LH and FSH after GnRH stimulating test are relatively high. This means that his leydig cells are not functioning well, suggesting that he may have hypergonadotropic hypogonadism and the possibility of delayed puberty in the near future. One previous literature has reported a 14-year-old boy presenting delayed puberty, so further follow-up is needed for our patient [11].

**Table 2:** Genotype–phenotype correlations in reported patients with mosaic 45,X/46,X,dic(Y)(q12)

Reference	Karyotype	Phenotype	Sex	Age (y)
Martin DesGroseilliers et al. [12], 2006	46,X,idic(Y)(q12)[226].ish idic(Y)(q12)(wcpY + ,SRY + + ,DYZ3 + +)/45,X[21]/47,X,idic(Y)(q12) × 2[3].ish idic(Y)(q12) × 2(wcpY + ,SRY + + ,DYZ3 + +) × 2	Dysmorphic features, mild language delay, small uterus	F	4
Willis, M. J. H et al. [13], 2006	45,X [14],/46,X, psu dic (Y)(q12)[5], (SRY + +)	normal	M	Infant
James Pascual et al.[14], 2009	45,X[8]/46,X,idic(Y)(q12)[12]	Ambiguous genitalia	M	Infant
Martin DesGroseilliers et al. [15], 2002	47,X,idic(Y)(q12) × 2[123]/45,X[9]	global psychomotor delay atrioseptal defect, radio-ulnar synostosis, bilateral fifth finger clinodactyly, premature closure of the anterior fontanel	M	2.3
Melanie Beaulieu Bergeron et al.[7], 2011	46,X,idic(Y)(q12)[138]/45,X[36]/46,X,del(Y)(q12)[1]	pure gonadal dysgenesis	F	unknown
Present study	45,X[8]/46,X,idic(Y)(q12)[32]	Short stature, mircopenis, hypospadias and cryptorchidism	M	8

The arrangement mechanism was studied before. An isodicentric Y chromosome is formed due to an aberrant homologous crossing over between opposite arms of a palindrome during spermatogenesis [16]. A duplication of the short arm and proximal long arm and a deletion of a part of the long arm is referred to as idic(Y)(q). The heterochromatin Yq12 region are unstable and fragile sites for breaking, that confer susceptibility to the formation of idic(Y)(q), possibly for the HSAT I/Alu/AT-rich tripartite repeat on human Yq12 is dramatic [17].

High-throughput sequencing technologies including WES, whole genome sequencing (WGS), CNV-Seq have been widely used in genetic diagnosis of rare diseases. In our study, whole Y chromosome mosaic duplication was firstly detected from CNV-Seq. However, the proportion of mosaics as well as the location of the breakpoints, which affect the clinical sex phenotype and disease severity, can not be determined, then cytogenetic tests was used to confirm the genetic diagnosis. Our case proved that the cytogenetic tests is still necessary technique for the patients detected as Y abnormality from sequencing because cytogenic tests can illuminate mosaics proportion and breakpoint location visually.

In conclusion, the present study reported the patient of a 10-year-old boy with mosaic 45,X/46,X,psu idic(Y)(q12). In addition to the common phenotypes, testicular biopsy of the boy showed no seminiferous tubules structure increasing a risk of delayed puberty and infertility. Our study proved that it would be beneficial to integrate high-throughput sequencing with cytogenetic technique for precise diagnosis, treatment and genetic counselling.

## Data Availability

The datasets used and analyzed during the current study available from the corresponding author on reasonable request.
